# Noninvasive Detection of Tuberculosis by Oral Swab Analysis

**DOI:** 10.1128/JCM.01847-18

**Published:** 2019-02-27

**Authors:** Angelique K. Luabeya, Rachel C. Wood, Justin Shenje, Elizabeth Filander, Cynthia Ontong, Simbarashe Mabwe, Hadn Africa, Felicia K. Nguyen, Alaina Olson, Kris M. Weigel, Lisa Jones-Engel, Mark Hatherill, Gerard A. Cangelosi

**Affiliations:** a South African Tuberculosis Vaccine Initiative (SATVI), Institute of Infectious Disease & Molecular Medicine and Division of Immunology, Department of Pathology, University of Cape Town, Cape Town, South Africa; b Department of Environmental and Occupational Health Sciences, School of Public Health, University of Washington, Seattle, Washington, USA; c Department of Anthropology, University of Washington, Seattle, Washington, USA; UNC School of Medicine

**Keywords:** GeneXpert MTB/RIF, molecular diagnosis, nonsputum sampling, oral swab, point of care, POC, PCR, tuberculosis

## Abstract

Diagnostic tests for tuberculosis (TB) usually require collection of sputum, a viscous material derived from human airways. Sputum can be difficult and hazardous to collect and challenging to process in the laboratory. Oral swabs have been proposed as alternative sample types that are noninvasive and easy to collect. This study evaluated the biological feasibility of oral swab analysis (OSA) for the diagnosis of TB. Swabs were tested from South African adult subjects, including sputum GeneXpert MTB/RIF (GeneXpert)-confirmed TB patients (*n* = 138), sputum GeneXpert-negative but culture-positive TB patients (*n* = 10), ill non-TB patients (*n* = 37), and QuantiFERON-negative controls (*n* = 34). Swabs were analyzed by using a manual, nonnested quantitative PCR (qPCR) targeting IS*6110*. Two swab brands and three sites within the oral cavity were compared. Tongue swabbing yielded significantly stronger signals than cheek or gum swabbing. A flocked swab performed better than a more expensive paper swab. In a two-phase study, tongue swabs (two per subject) exhibited a combined sensitivity of 92.8% relative to sputum GeneXpert. Relative to all laboratory-diagnosed TB, the diagnostic yields of sputum GeneXpert (1 sample per subject) and OSA (2 samples per subject) were identical at 49/59 (83.1%) each. The specificity of the OSA was 91.5%. An analysis of “air swabs” suggested that most false-positive results were due to contamination of manual PCRs. With the development of appropriate automated methods, oral swabs could facilitate TB diagnosis in clinical settings and patient populations that are limited by the physical or logistical challenges of sputum collection.

## Abstract (precis)

Diagnostic tests for tuberculosis (TB) usually require collection of sputum, a viscous material derived from human airways. Sputum can be difficult and hazardous to collect and challenging to process in the laboratory.

## INTRODUCTION

There is a significant need for the early detection and treatment of patients with active pulmonary tuberculosis (TB) in order to prevent the transmission of the causative agent Mycobacterium tuberculosis (1). Most current diagnostic tests for TB rely on sputum sampling from symptomatic TB patients (2–5). Sputum sampling has limitations in young children and in other patients who are unable to expectorate and in patients with paucibacillary disease. Sputum sample quality can be variable even from adult patients with productive coughs (6). Sputum collection produces potentially infectious aerosols, a hazard for health care workers and fellow patients. These challenges are amplified in active case-finding scenarios that require high-throughput sampling of large numbers of people (7–10).

Easier, safer, and more effective sampling methods for TB are needed (11). Recent studies have evaluated alternative samples, such as saliva, urine, blood, and exhaled breath concentrate (3, 12–15). Unfortunately, these samples typically had lower sensitivity or specificity than sputum.

Our consortium previously evaluated oral (buccal) swabs as alternative nonsputum samples (16). Oral swabbing has been used to detect TB in nonhuman primates that do not produce sputum (17, 18), and mycobacteria are known to associate with diverse biological and environmental surfaces (19–23). Therefore, we hypothesized that bacilli that pass through the mouths of TB patients accumulate on oral epithelia in sufficient quantities for detection by quantitative PCR (qPCR). Three swabs per subject were collected from 20 GeneXpert-confirmed TB cases in South Africa and from age-matched healthy controls in the United States. In total, 18 of the 20 GeneXpert-confirmed case subjects (90%) yielded positive swabs. One-hundred percent of U.S.-based healthy control subject swabs were negative (16).

Oral swabbing is very easy to perform. Collection is painless, noninvasive, and nonaerosol producing. In contrast to many alternative TB sample types (3, 12–15), swabbing takes only seconds to complete and does not require privacy or isolation. Self-sampling at home is routine in direct-to-consumer genetic testing (24). A study of Wood et al. (16) yielded encouraging results for oral swabs, but the numbers were small, it was not carried out in a blind manner, and healthy controls were not recruited from the same population as the cases.

The present study had two goals. First, it evaluated alternative sampling approaches, including a less expensive swab product and alternative swab sites within the mouth. Second, oral swab analysis (OSA) was evaluated in a larger study than the previous one (16), was carried out in a partial blind manner, and used cases and controls that came entirely from a community in South Africa where TB is endemic. The results further support the biological feasibility of OSA, in that M. tuberculosis DNA and/or cells were detected in the mouths of >90% of sputum GeneXpert-positive TB patients.

Because existing molecular diagnostic tests, such as the GeneXpert MTB/RIF (Cepheid Inc., Sunnyvale, CA), are engineered for sputum analysis, not swab analysis, the current study used a manual qPCR designed specifically for testing swab samples. The results confirmed that M. tuberculosis DNA is commonly present in the oral cavities of TB patients. This finding sets the stage for the development and evaluation of automated methods designed to detect M. tuberculosis DNA in oral swab samples.

## MATERIALS AND METHODS

### Study site and population.

**FIG 1:**
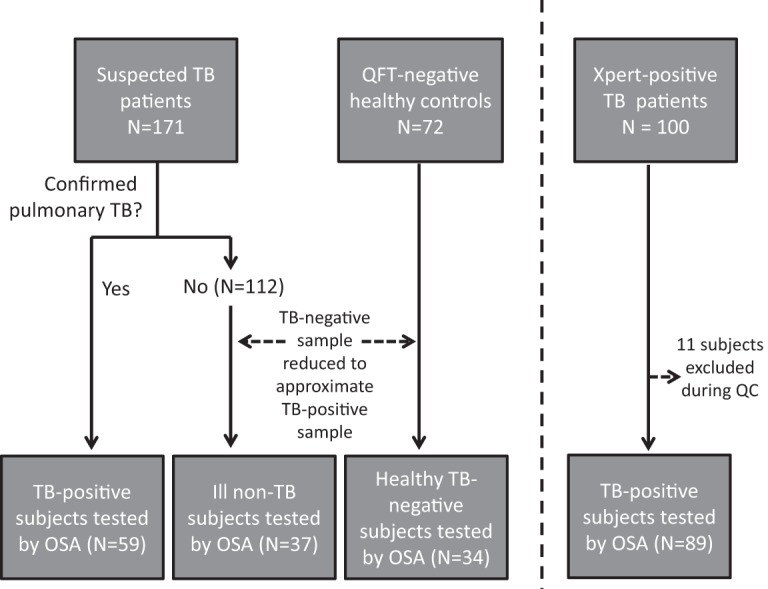
Flow diagram of subject enrollment and testing by OSA. Total subjects enrolled in the study (combined *n* = 343) are shown in the top row of boxes. The bottom row shows subjects tested by OSA (combined *n* = 221).

Worcester is a semirural region in Western Cape, South Africa, with a population of around 350,000 people. Subjects were referred to the clinical site by directly observed treatment, short-course (DOTS) or health care providers or were self-referred when symptoms became severe or chronic. Subjects were recruited into the present study in two phases (Fig. 1). In phase 1, oral swabs were collected from patients with suspected TB (*n* = 171) along with QuantiFERON (QFT-GIT)-negative healthy controls (*n* = 72). TB-negative subjects in phase 1 (QFT-negative controls and ill non-TB cases) outnumbered TB-positive subjects by 3-to-1 (Fig. 1). Therefore, a subset of 71 TB-negative subjects was selected for manual qPCR analysis of swabs, based on date of enrollment (the first consecutive TB-negative subject enrolled after each phase 1 TB-positive subject was selected). This strategy was used to prevent bias and to help ensure that TB-negative samples were collected on nearly the same dates as TB-positive samples.

In phase 2, swabs were collected from 100 GeneXpert-confirmed TB patients. Of these patients, 11 were subsequently excluded because of mislabeling noted during quality control, leaving 89 sputum GeneXpert-confirmed TB cases (Fig. 1, phase 2). Phase 2 expanded the total number of confirmed TB cases in the study and tested a method enhancement that was hypothesized based on phase 1 results.

### Phase 1 QFT-negative controls.

Healthy adult participants aged ≥18 years old with negative QFT results (*n* = 72) were screened and recruited as controls from ongoing studies at the South African Tuberculosis Vaccine Initiative (SATVI).

### Phase 1 patients with suspected TB.

Adult patients (≥18 years old) were approached and gave their consent at the TB clinic when they visited for possible TB (*n* = 171). Initially, symptoms required for enrollment were at least 3 of the following: productive cough, fatigue, night sweats, fever, unexplained cough for more than 3 weeks, unexplained weight loss, chest pain, and hemoptysis. During the course of the study, a more restricted approach for inclusion was implemented in which potential subjects had to have at least 3 of the following: productive cough, unexplained weight loss, chest pain, and hemoptysis. This change was designed to increase the screening sensitivity so that a greater proportion of suspected cases would have microbiologically confirmed disease.

For all subjects, the first (day 1) oral swab was collected at the clinic before the sputum sample was collected for GeneXpert testing. Two subsequent sampling sessions occurred on separate days. Day 2 and day 3 samples were collected by study personnel either at subjects’ homes or in the clinic. All subjects were instructed not to brush their teeth, use mouthwash, eat, or drink 30 min before giving the swab sample on the second and third swabbing sessions, which occurred mostly in the early morning. No swabs were collected immediately after sputum collection.

The phase 1 cohort of 171 TB suspects included 112 ill non-TB patients (Fig. 1). These patients presented symptoms suggestive of TB disease but had negative sputum GeneXpert MTB/RIF results and no other indications of pulmonary TB. All ill non-TB patients had an additional sputum sample collected that was sent to BARC Global Central Laboratory in Johannesburg (BARC South Africa Pty, Ltd.) for liquid culture (mycobacterial growth indicator tube [MGIT] Becton, Dickinson) to detect M. tuberculosis. If a positive culture result was returned, that patient was moved into the TB-positive category. The remaining ill non-TB cases were followed up with monthly clinical assessments for 1 year and were moved out of the ill non-TB category if TB was diagnosed during this period. Accordingly, the definition for ill non-TB was the following: two negative sputa (one tested by GeneXpert and the other by culture) and recovery from illness without TB treatment.

The phase 1 cohort was found to include 59 TB-positive cases, defined as patients with the symptoms outlined above, combined with either a positive sputum GeneXpert (*n* = 49) or a positive MGIT culture result (*n* = 10) (Fig. 1). All TB cases underwent TB treatment.

### Oral swab collection.

In Phase 1, 2 cheek swabs (1 from each cheek), 1 tongue swab, and 1 gum swab were collected at each of the 3 sessions, yielding 12 swabs from each subject (4 per day). The tongue swab, gum swab, and one cheek swab were Whatman OmniSwabs (catalog number WB100035), the same type of swab used in the previous study (16). The other cheek swab was a Puritan PurFlock Ultra swab (catalog number 25-3606-U).

Cheek swabs were collected as described previously (16). Tongue swabs were collected from the dorsum of the tongue, taking care not to reach far back into the mouth. For all swabs, the study staff firmly brushed the swab along the appropriate surface 7 to 8 times (about 10 s total) each. After swabbing, the head of the swab was ejected (OmniSwab) or snapped off (PurFlock) into a tube containing sterile antimicrobial lysis buffer (500 µl), as described previously (16).

In addition to oral swabs, control swabs (“air swabs”) were collected by exposing swabs in the air for 10 s at the sampling place. Air swabs were put into a tube containing sterile lysis buffer and processed alongside oral swabs.

In both study phases, 3 sessions of oral swab sampling were performed on each subject on 3 separate days. All swabs were collected within 5 days after any treatment was initiated. All samples (500 µl lysis buffer with swab head) were stored at −80°C within 8 h of collection and analyzed in batches. Storage times at −80°C ranged from 1 to several months.

### Phase 2-confirmed TB cases.

In order to increase the number of confirmed TB cases in the study and to extend swab brand comparisons, we enrolled an additional cohort of 100 sputum GeneXpert-confirmed TB cases. Swabs from 89 of these cases were tested by OSA (Fig. 1, phase 2). Phase 2 samples consisted solely of tongue swabs. OmniSwabs were used on the first and third swabbing session in phase 2, and a PurFlock Ultra swab was used in the second swabbing session. The timing and methods of sample collection were the same as phase 1, with the exception that the first sample was collected after TB diagnosis.

### DNA extraction.

The laboratory team was blind to the TB status of subjects in phase 1 but not in phase 2. DNA was extracted from swab samples by using the spin column protocol of the Qiagen QIAamp DNA minikit (catalog number 51306). Before opening the tube, each sample was heated to 95°C for 10 min in a floating rack in a water bath to inactivate pathogens for safe laboratory processing. The spin column protocol described previously for buccal swabs (16) was slightly modified to increase the DNA yield into the eluate. To accomplish this, the DNA elution step was completed twice using 150 µl of Buffer AE with an extended incubation time of 3 min at room temperature and 3 min at 42°C. In preparation for PCR, the dried DNA pellet resulting from ethanol precipitation was resuspended in 15 µl of 3:1 molecular grade water and Buffer AE, rather than 5 µl of Buffer AE, as described previously (16). This change was made to increase the efficacy of resuspension and to enable a shorter resuspension time of 15 min. The entire 15-μl volume was added to a concentrated PCR master mix.

### qPCR analysis.

The nonnested qPCR protocol was as described previously (16), except that the master mix recipe was adjusted for the increased DNA resuspension volume. A threshold *C_q_* value of 38 was used to determine OSA positivity. The threshold was derived from a preliminary analysis of air swabs. Primers in this reaction were designed to amplify IS*6110*, a multicopy insertion element unique to the M. tuberculosis complex (25, 26). Every qPCR run included a positive control consisting of a healthy volunteer oral swab “spiked” with cultured M. tuberculosis H37Ra DNA and a negative control consisting of a sterile swab. All results were rejected when either control failed.

### Clinical data.

We collected sociodemographic information, including participant’s age, gender, ethnicity, smoking and alcohol intake status, area of residence, level of education, employment status, and recent exposure to household TB contacts. Clinical symptoms in the last 2 weeks were also recorded and included chest pain, cough, fatigue, loss of weight, fever, night sweats, and hemoptysis. Chronic conditions, such as HIV infection or diabetes mellitus, were recorded.

### Statistical analysis.

Stata 11 (StataCorp LLC) was used for the analysis. A paired *t* test was used to compare intrasubject continuous variables, namely mean qPCR signals (*C_q_* values). An unpaired *t* test was used to compare continuous variables by group defined by a categorical variable. Paired z tests were used to compare percent sensitivities between methods. Tests were 2-tailed unless otherwise stated. Linear regression models were generated to analyze the association between clinical characteristics and mean qPCR signals. Confidence intervals of proportions were 95%. A 0.05 significance level was used for all statistical analyses.

## RESULTS

### Subject population.

In total, 343 participants were enrolled in phases 1 and 2. Among them, 159 were laboratory-confirmed TB cases, 72 were QFT-negative healthy participants, and 112 were suspected TB patients who were determined not to have pulmonary TB (ill non-TB) (Fig. 1). Participant characteristics are presented in Tables S1 and S2 in the supplemental material.

The phase 1 cohort included 59 laboratory-confirmed TB cases. Sputum GeneXpert results were positive for 49 of these 59 cases (83.1%). The 10 GeneXpert-negative patients were diagnosed with TB disease by sputum MGIT culture result. As outlined in the Materials and Methods, TB-negative subjects greatly outnumbered TB-positive subjects in phase 1, so swabs from 34 QFT-negative subjects and 37 ill non-TB subjects were selected for analysis (totaling 71 TB-negative subjects in phase 1). Day 1 and day 2 swabs were tested in this analysis; day 3 swabs were reserved for future studies.

### Tongue swabs yielded stronger signals than cheek or gum swabs.

Signals by qPCR were stronger in tongue OmniSwabs than in cheek OmniSwabs or gum OmniSwabs. Cheek OmniSwabs were used in the previous study (16). Table 1 compares mean *C_q_* values from the three oral sites. *C_q_* values are measures of qPCR signal strength based on amplification cycle number; smaller values are stronger signals. Tongue OmniSwabs from GeneXpert-positive subjects yielded signals that averaged 5.4 *C_q_* values lower than cheek OmniSwabs collected from the same mouths at the same time (day 1 and day 2 swabs from 59 GeneXpert-positive subjects; *P* < 0.0001 in paired *t* test). This corresponds to ∼48-fold stronger signal in tongue swabs. Gum OmniSwabs performed less well than cheek OmniSwabs (Table 1). When comparing swab brands, cheek PurFlock swabs yielded slightly stronger signals than cheek OmniSwabs (*P* = 0.015) (Table 1).

**TABLE 1:** Comparison of alternative swabbing sites and swab brands

Site and brand	Mean *C_q_* ± SD (*n* = 118)	Significance relative to cheek OmniSwab (*P* value, paired *t* test)
Cheek, OmniSwab^*a*^	38.8 ± 5.3	—^*b*^
Tongue, OmniSwab	33.4 ± 6.8	<0.0001
Gum, OmniSwab	39.6 ± 5.1	0.019
Cheek, PurFlock	37.9 ± 6.0	0.015

a Site and brand used in an earlier study (16).

b —, Not applicable, reference method.

### Sensitivity, specificity, and diagnostic yield of OSA within the phase 1 cohort.

As tongue swabs yielded the strongest qPCR signals, we focused our analysis on tongue swabs collected on days 1 and 2. At least 1 positive swab was observed in 45/49 sputum GeneXpert-positive TB cases (91.8%) in phase 1 (Table 2). OSA was also positive for 4 of the 10 cases who were negative by sputum GeneXpert but diagnosed by sputum culture.

**TABLE 2:** Sensitivities and specificities of OSA in phase 1

Sampling method	No. (%) of positive swabs, indicating:		
	Sensitivity relative to sputum Xpert MTB/RIF (*n* = 49)	Sensitivity relative to all TB cases (*n* = 59)	Specificity relative to ill non-TB and healthy controls (*n* = 71)
Sputum, GeneXpert MTB/RIF	NA^*a*^	49/59 (83.1)	ND^*b*^
OSA, 2 swabs/subject, at least 1 swab positive	45/49 (91.8)	49/59 (83.1)	65/71 (91.5)
OSA, 1 swab on Day 1	39/49 (79.6)	42/59 (71.2)	67/71 (94.4)
OSA, 1 swab on Day 2	42/49 (85.7)	46/59 (78.0)	69/71 (97.2)

a NA, not applicable.

b ND, not determined.

Of 71 QFT-negative and ill non-TB subjects, OSA generated true negative results (both tongue swabs negative) for 65 subjects (91.5% specificity) (Table 2). The 71 TB-negative subjects provided 2 swabs each, for a total of 142 individual tongue swabs. Among these, 136 swabs (95.8%) yielded negative results. An equivalent number of air swabs were also tested. Air swab specificity was 135/140 (96.4%). Therefore, at least some of the background signal seen in tongue swabs may have resulted from contamination during collection or manual analysis.

Relative to all TB-positive subjects, 49/59 (83.1%) had at least 1 positive swab out of 2 swabs tested. This yield was identical to that of a single sputum GeneXpert within this cohort (Table 2). The two methods exhibited comparable yields because each detected 4 cases that the other did not. Single-swab yields were 42/59 (71.2%) on day 1 and 46/59 (78.0%) on day 2. These yields were smaller than the 83.1% yield of the single sputum test, although the differences were not statistically significant (*P* > 0.05).

### Timing of sample collection.

Within the phase 1 cohort, day 2 tongue swabs yielded stronger M. tuberculosis qPCR signals than day 1 tongue swabs (*P* = 0.013; Table 3). The reason is not known, but the time of day of sample collection might have been relevant. Day 1 sample collection occurred throughout the day, depending on when patients were seen at the clinic. In contrast, most day 2 samples were collected in the early morning (Table 3).

**TABLE 3:** Comparison of tongue swabs

Parameter	Day 1	Day 2
Mean *C_q_* ± SD^*a*^	34.3 ± 6.6	32.6 ± 6.9
Sensitivity relative to all TB (*n* [%])	42/59 (71.2)	46/59 (78.0)
Collection time (range)	5:30 a.m.–3:25 p.m.	4:00 a.m.–11:35 p.m.
Collection time (median)	10:45 a.m.	7:00 a.m.

a Significant by paired *t* test (*P* = 0.013).

### Phase 2: expansion of TB-positive sample set and comparison of tongue swab brands.

Phase 1 provided evidence that PurFlock Ultra swabs yielded a stronger signal than OmniSwabs, in the context of cheek swabbing (Table 1). Although the difference was modest, even equivalent signals are noteworthy because PurFlock Ultra swabs are less expensive than OmniSwabs ($0.36 versus $1.67, respectively). To ask whether the same is true in the context of tongue swabbing, we analyzed both brands from 89 additional sputum GeneXpert-positive TB patients (Fig. 1). This cohort also increased our number of GeneXpert-positive subjects for sensitivity calculations.

Procedures followed those in phase 1, except that the study was not conducted in a blind manner and subjects were enrolled after they had tested positive by sputum GeneXpert. Two tongue swabs were tested per subject; an OmniSwab was collected on day 1 and a PurFlock Ultra collected on day 2 (two subjects had swab samples on 1 day only).

At 93.3%, the sensitivity of two swabs relative to a single sputum GeneXpert was comparable to that of phase 1. The PurFlock brand averaged 3 *C_q_* values lower (corresponding to ∼8-fold stronger signal) than OmniSwabs (Table 4). A caveat is that day 2 samples yielded stronger signals than day 1 samples in phase 1, even when the same swab brand was used (Table 3). Although the difference between the 2 days was modest, it could account for at least a portion of the apparent advantage of PurFlocks seen in Table 4.

**TABLE 4:** Comparison of day 1 OmniSwabs to day 2 PurFlock swabs in phase 2

OSA	Mean *C_q_* ± SD	No. (%) of positive swabs, indicating sensitivity relative to sputum GeneXpert
1 OmniSwab on day 1	33.5 ± 5.7	74/89 (83.1)
1 PurFlock on day 2	30.5 ± 6.5^*a*^	74/87 (85.1)
2 swabs/subject, at least 1 positive	NA^*b*^	83/89 (93.3)

a Significantly different from day 1 OmniSwab (*P* < 0.0005 in paired *t* test).

b NA, not applicable.

Combining phase 1 and phase 2, the sensitivity of tongue swabbing (2 samples per subject) relative to sputum GeneXpert (1 sample per subject) was 128/138 (92.8%).

### Relationship between OSA signal and HIV coinfection.

Among TB patients tested by OSA in phase 2, 34 were coinfected with HIV and 55 were not. The mean *C_q_* signal in HIV-coinfected TB cases was higher (weaker) than in non-HIV-infected patients (Table 5).

**TABLE 5:** Comparison of HIV-coinfected and noninfected subjects in phase 2

Day of collection	HIV-coinfected subjects (*C_q_* ± SD) (*n* = 34)	HIV-noninfected subjects (*C_q_* ± SD) (*n* = 55)	*P* value (*t* test)
1	35.8 ± 6.9	33.1 ± 5.2	0.037
2	33.5 ± 6.9	30.1 ± 6.7	0.034

## DISCUSSION

We evaluated OSA for the detection of TB in a high prevalence setting. Two swab brands and three sites in the oral cavity were compared. Overall, 2 oral swabs per patient were as sensitive as a single sputum GeneXpert test. Because sputum GeneXpert was a reference method used to classify subjects as TB patients and because it was not applied to QFT-negative healthy controls, our study did not compare the specificities of oral swabs and sputum testing.

Many patients struggle to produce adequate sputum for testing, especially in active case-finding scenarios (7–10). It is for these situations that easy-to-collect, non-invasive sputum alternatives are needed (11). Although OSA detected only 92.8% of sputum GeneXpert-positive cases in phases 1 and 2 combined, the diagnostic yields of two swabs and one sputum relative to all TB were similar. Single-swab yields were slightly below that of a single sputum test. Further development of OSA may close this performance gap. In the meantime, OSA may find its greatest utility in situations that are limited by the physical or logistical challenges of sputum collection.

We suspect that M. tuberculosis cells or DNA are deposited nonspecifically on oral surfaces. In phase 1, tongue OmniSwabs from TB cases yielded stronger signals than cheek or gum OmniSwabs. Relative to buccal surfaces, the lingual papillae that give the tongue its rough texture may be better at entrapping bacilli that pass through the mouth over the normal course of exhalation, coughing, and sputum production in active TB.

Oral swabs collected on day 2 yielded stronger qPCR signals than samples collected on day 1. Day 2 samples were on average collected earlier in the day. This observation might reflect circadian rhythms in sputum positivity or salivary flow (27). Alternatively, daytime activities, such as eating, drinking, and oral hygiene, might diminish M. tuberculosis analytes on oral surfaces. It is also possible that days 1 and 2 differed for reasons other than time of day of sample collection.

This study used a manual qPCR protocol designed specifically for this novel sample type. At 91.5% by subject, the specificity of OSA was less than optimal. On a swab-by-swab basis, the false positivity rate of 4.2% (6 out of 142 swabs) was nearly identical to that of air swabs collected on-site (5 out of 140 swabs or 3.6%). Our manual protocols may have provided opportunities for sample contamination that might not exist if automated platforms are used. Although the volume sampled by air swabs was very small, they may also have been contaminated during exposure to air in the clinic or at patients’ homes.

As diagnostic samples, swabs differ significantly from sputum. They may have fewer bacilli on average than sputum, but they are also smaller in volume, less viscous, less complex, and associated with a solid support. Additional work is needed to adapt OSA for use on automated diagnostic platforms, such as GeneXpert MTB/RIF, which are specifically designed for processing sputum, not swabs. The results of the current biological feasibility study make a case for the development of such adaptations.

qPCR signals were weaker in the HIV-infected than in the non-HIV-infected TB patients. In this way, OSA resembles many other TB diagnostic methods. HIV-coinfected TB patients tend to have reduced M. tuberculosis bacillary load in expectorated sputum (6).

The current study improved upon the previous one (16) by including negative controls recruited from the local South African population. However, TB patients and non-patients were not perfectly matched in all demographic characteristics. Overall, participants in the QFT-negative healthy control group were younger, less educated, more likely to be female, and less likely to be black Africans than confirmed TB and ill non-TB subjects (Table S1).

Additional limitations of the current study include a small sample size, a single geographical region, and the fact that phase 2 was not conducted in a blind manner. Our case definition in phase 1 required 1 positive sputum GeneXpert result or 1 positive sputum culture, consistent with the standard of care in South Africa. The study would have been more robust if positive culture was required to define all cases. However, the first-generation GeneXpert is >98% specific relative to culture. Therefore, if there were false positives among the cases in either phase, they would likely have been very few in number (<2%).

An additional limitation was the use of manual qPCR, which may have decreased specificity. Moreover, children were not included. Larger, multisite studies of OSA, involving all ages and using automated swab analysis methods, are needed.

Despite these limitations, the results confirm and significantly expand our previous finding (16) that M. tuberculosis DNA and/or cells accumulate in the oral cavities of TB patients in amounts that are sufficient to enable non-sputum-based diagnosis of TB in at least some patients.

## Supplementary Material

Supplemental file 1
