# Supplementary material for: Noninvasive Detection of Tuberculosis by Oral Swab Analysis
Source: J Clin Microbiol. 2019 Feb 27;57(3):e01847-18. doi: 10.1128/JCM.01847-18 (PMC6425180; doi:10.1128/JCM.01847-18)
Supplement: Supplemental file 1 [file JCM.01847-18-s0001.pdf]

Luabeya AK et al. “Non-invasive detection of tuberculosis by oral swab analysis”

Supplemental Data: Demographic and health characteristics of study populations

Table S1. Socio-demographic characteristics of participants

	Healthy controls N=72	TB patients N=159	Ill non-TB patients N=110	p*
**Age in years**				
Mean (SD)	21.28 (6.87)	36.4 (11.9)	37.3 (13.1)	<0.001
Median (IQR)	18.8 (18.3-19.6)	35.2 (25.6-45.8)	36.3 (26.3-45.9)	
**Gender (%)**				0.004
Male	26 (36.1)	91 (57.2)	46 (41.8)	
Female	46 (63.9)	69 (42.8)	64 (58.1)	
**Ethnicity (%)**				0.002
Black	25 (34.7)	79 (49.7)	59 (54.6)	
Mixed race	44 (61.1)	80 (50.3)	51 (46.4)	
White	3 (4.3)	0	0	
**Employed (%)**	16 (22.2)	68 (42.7)	52 (47.2)	0.002
**Education level (%)**				<0.001
Primary	0	48 (30.2)	30 (27.2)	
Post primary	72 (100)	111 (69.8)	80 (72.7)	
**Smoker (%)**	18 (25.0)	94 (59.1)	58 (53.7)	<0.001
**Drinking Alcohol (%)**	23 (31.9)	74 (46.5)	48 (43.6)	0.1
**Housing type (%)**				<0.001
House	58 (80.6)	81 (50.9)	53 (48.2)	
Flat	4 (5.6)	6 (3.7)	10 (9.1)	
Farm	0	1 (0.6)	1 (0.9)	
Informal settlement	10 (13.9)	71 (44.7)	46 (41.8)	

*p value, patients vs. healthy controls

Table S2. Clinical characteristics of patients enrolled in the study

	TB patients N=159 N (%)	Ill-non TB patients N=110 N (%)	P value*
Cough for 2 weeks	148 (93.1)	105 (95.5)	0.42
Chest pain	121 (76.1)	92 (83.6)	0.13
Fatigue	46 (28.9)	19 (17.2)	0.03
unexplained fever	100 (62.7)	72 (64.5)	0.67
Hemoptysis	5 (3.1)	7 (6.3)	0.21
Breath shortness	97 (61.0)	53 (48.2)	0.04
Night sweats	129(81.3)	93 (84.5)	0.47
Loss of weight	141 (88.7)	86(78.1)	0.02
HIV infection (N with data available)	60 (38.0) (N=158)	26 (24.3) (N=107)	0.02
Diabetes Mellitus	6 (3.7)	2 (1.9)	0.35
Household TB contact	23 (14.5)	14 (12.7)	0.68
Previous TB disease	25 (16.0)	1 (0.9)	<0.001
